# Nutrient-Deprivation Autophagy Factor-1 (NAF-1): Biochemical Properties of a Novel Cellular Target for Anti-Diabetic Drugs

**DOI:** 10.1371/journal.pone.0061202

**Published:** 2013-05-22

**Authors:** Sagi Tamir, John A. Zuris, Lily Agranat, Colin H. Lipper, Andrea R. Conlan, Dorit Michaeli, Yael Harir, Mark L. Paddock, Ron Mittler, Zvi Ioav Cabantchik, Patricia A. Jennings, Rachel Nechushtai

**Affiliations:** 1 The Alexander Silberman Institute of Life Science, Hebrew University of Jerusalem, Edmond J. Safra Campus at Givat Ram, Jerusalem, Israel; 2 Departments of Chemistry and Biochemistry and Physics, University of California San Diego, La Jolla, California, United States of America; 3 Department of Biology, University of North Texas, Denton, Texas, United States of America; Weizmann Institute of Science, Israel

## Abstract

Nutrient-deprivation autophagy factor-1 (NAF-1) (synonyms: Cisd2, Eris, Miner1, and Noxp70) is a [2Fe-2S] cluster protein immune-detected both in endoplasmic reticulum (ER) and mitochondrial outer membrane. It was implicated in human pathology (Wolfram Syndrome 2) and in BCL-2 mediated antagonization of Beclin 1-dependent autophagy and depression of ER calcium stores. To gain insights about NAF-1 functions, we investigated the biochemical properties of its 2Fe-2S cluster and sensitivity of those properties to small molecules. The structure of the soluble domain of NAF-1 shows that it forms a homodimer with each protomer containing a [2Fe-2S] cluster bound by 3 Cys and one His. NAF-1 has shown the unusual abilities to transfer its 2Fe-2S cluster to an apo-acceptor protein (followed *in vitro* by spectrophotometry and by native PAGE electrophoresis) and to transfer iron to intact mitochondria in cell models (monitored by fluorescence imaging with iron fluorescent sensors targeted to mitochondria). Importantly, the drug pioglitazone abrogates NAF-1's ability to transfer the cluster to acceptor proteins and iron to mitochondria. Similar effects were found for the anti-diabetes and longevity-promoting antioxidant resveratrol. These results reveal NAF-1 as a previously unidentified cell target of anti-diabetes thiazolidinedione drugs like pioglitazone and of the natural product resveratrol, both of which interact with the protein and stabilize its labile [2Fe-2S] cluster.

## Introduction

NAF-1 (Nutrient-deprivation autophagy factor-1, synonyms: ERIS, Miner1) is a member of a newly discovered family of iron-sulfur (FeS) proteins coded by *Cisd* genes that are defined by a unique CDGSH amino acid sequence in their FeS cluster binding domain [1]–[4]. Interest in NAF-1 has recently increased because the gene is in a region of chromosome associated with neuronal development [5], and it is now known to be critical for the maintenance of skeletal muscle [6] and for promoting longevity [7],[8]. Moreover, a transcriptional splicing error leads to a rare but serious disease called Wolfram Syndrome 2 [9], which is associated with hearing deficiencies, severe blindness and diabetes and a lower life expectancy. The protein, which is localized in both ER [9] and in mitochondria [7], has been functionally implicated in cell autophagy, possibly as a mediator of Bcl-2 antagonism of Beclin-1 dependent autophagy on the surface of the ER [10].

**Figure 1:**
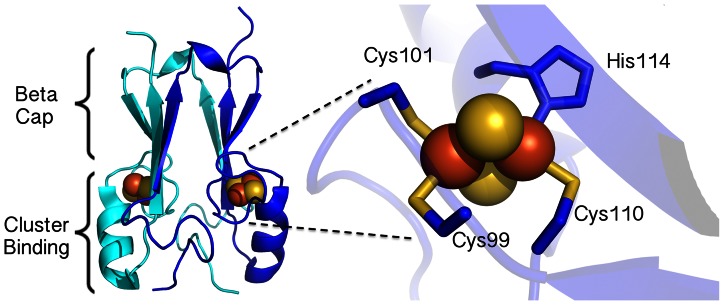
Structure of NAF-1. (*A*) Ribbon diagrams of the soluble parts of NAF-1 (amino acids 57–135) derived from published X-ray analyses (1) (PDB code 3FNV). The protein is homodimeric and is comprised of two main domains – the beta cap and the cluster binding domain, where each protomer contains a [2Fe-2S] cluster (sulfur and iron depicted as yellow and orange spheres, respectively). (*B*) A magnification of one of the NAF-1 cluster binding sites highlighting the single-coordinating His and three coordinating Cys residues.

The crystal structure of NAF-1 [1] showed that it is a homodimeric [2Fe-2S] protein and each protomer harbors one 2Fe-2S cluster bound to the protein by an usual 3-Cys-1-His coordination geometry (Fig. 1). The structure bears a similar backbone fold [1] to its paralog mitoNEET (Fig. S1) [2], a previously identified target of the thiazolidinedione (TZD) class of anti-diabetes drugs [11],[12]. Recent work has suggested that there is an additional mitochondrial target for this class of drug [13] so we sought to determine if NAF-1 was a bona fide target of TZDs. Importantly, these results have bearing on the development of alternative treatments for type II diabetes as until now, the pharmacological (both beneficial and deleterious) effects of the TZD drugs have been widely linked to the peroxisome proliferator-activated receptor gamma PPARγ [11]–[16].

As we have recently assigned a functional role for mitoNEET as a cluster transfer protein whose iron transfer into mitochondria is abrogated by TZD drugs [17], we investigated the cluster transfer ability of NAF-1. Transfer from NAF-1 to apo-acceptors was found to only occur when the [2Fe-2S] clusters were oxidized ([2Fe-2S]^2+^ state) and the free cysteines of the acceptor protein were reduced (free thiol form). The addition of glutathione (GSH), a physiological reducing agent, was effective in preparing the apo-acceptor protein for cluster transfer. Addition of NAF-1 to cells leads to iron accumulation in mitochondria. The anti-type II diabetes drug pioglitazone stabilized NAF-1 [2Fe-2S] clusters from release and prevented iron overload in mitochondria. Replacement of the single His ligand to the 2Fe-2S cluster with Cys stabilized the cluster, inhibited cluster transfer to apo-acceptor proteins and inhibited iron transfer into the mitochondria. These effects are similar to those previously found for mitoNEET [17] and suggest that NAF-1 could be a bona fide mitochondrial target of TZDs. However, interestingly, the anti-diabetic and longevity promoting natural product resveratrol showed a similar effect on cluster stabilization and inhibition of iron overload specifically for NAF-1. These results suggest a formerly unrecognized and important role for NAF-1 in responding to oxidative stress, which is known to be a major contributor to the onset of type II diabetes and act as a predominant cause of aging.

## Experimental Procedures

All the materials used in this work were from best available commercial grade. The mitochondrial iron sensor red rhodamine B-[(1,10-phenanthrolin-5-yl) aminocarbonyl] benzyl ester (RPA) was obtained as described elsewhere [18]. Pioglitazone (Bosche Scientific, New Brunswick, NJ) was dissolved in 100% DMSO and diluted in test medium prior to use in cell assays. Resveratrol (Cayman Chemical, Ann Arbor MI) was dissolved in 100% ethanol and diluted in test medium prior to use in cell assays.

### Expression and purification of NAF-1 proteins and Ferredoxin

The human NAF-1 cDNA encoding the cytoplasmic soluble (NAF-1) part of the protein (residues 57–135) was amplified by PCR and subcloned into a modified pET28-a (+) vector (Novagen, Madison, WI) as described [1]. Purification of NAF-1 and the H114C mutant was performed as described previously [1]. Expression and purification of ferredoxin (Fd) in both holo- and apo-forms was performed as described previously [19].

### UV-Vis absorption spectroscopy, transfer kinetics, and decays

Absorption spectra were recorded at 350–600 nm (CARY, 300Bio), equipped with a temperature control apparatus set to 37°C. Special attention was given to changes in absorbance at 458 nm (NAF-1's signature [2Fe-2S] absorbance peak) and at 423 nm (characteristic of the [2Fe-2S] cluster in Fd). The % of cluster transfer CT(%) at any given time was determined from the ratio A^423^/A^458^ values as follows: CT(%) = (ΔR/ΔR_max_)×100, where ΔR = R_obs_−R_init_ and ΔR_max_ = R_final_−R_init_. R_obs_ is the observed A^423^/A^458^ ratio at a given time, R_init_ is the initial A^423^/A^458^ ratio observed at time 0, which is equal to 0.85, and R_final_ is the A^423^/A^458^ ratio at long times when the reaction is considered complete and equal to 1.14. Data is normalized and fit to a single exponential decay. Initial transfer rates were determined by taking the tangent of the slope of the fit early into the transfer process (10 min) when concentrations of NAF-1 and apo-Fd were still close to their starting amounts. Kinetic measurements were performed using equimolar concentrations of NAF-1 (WT and H114C) and apo-Fd in the presence of 50 mM Tris pH 8.0, 100 mM NaCl, 5 mM DTT, and 5 mM EDTA, unless stated otherwise. The apo-Fd and DTT were pre-incubated for 30 min prior to the start of the experiment. Decays were performed at 37°C and determined by monitoring loss of the 458-nm peak with time. Data were then fit to a single exponential rise. Studies were performed using varying concentrations of NAF-1 in 100 mM Bis-tris 100 mM NaCl for pH 7.0.

### Native-PAGE [2Fe-2S] cluster transfer in vitro assay

NAF-1 and the H114C mutant were incubated with apo-Fd. NAF-1 and apo-Fd concentrations were 200 μM and 400 μM, respectively, so that bands could be clearly visualized. The NAF-1 and apo-Fd were incubated under vigorous aeration in the presence of 100 mM DTT or 2% β-mercaptoethanol and 5 mM EDTA for the specified time lengths (0–60 min). Transfer of the [2Fe-2S] cluster from NAF-1 to apo-Fd was then analyzed by native-PAGE [17] and then stained with Coomassie to show presence of both proteins.

### Cell culture

Human h9c2 cells were grown at 37°C in 5% CO_2_ in Dulbecco's modified Eagle's medium (DMEM; Biological Industries) supplemented with 1% antibiotics (penicillin, streptomycin, and amphotericin), 1% glutamine, and 10% fetal calf serum.

### Fluorescence measurements

H9c2 cells were seeded to an optimal density of 1–2 million cells pre plate – 3 cm perforated with a microscopic slide attached. The cells were labeled for 15-min at 37°C with 1 μM RPA in DMEM medium containing 10 mM HEPES buffer instead of phenol red and supplemented with 10 μM desferrioxamine (DFO) (Sigma-Aldrich) to prevent quenching of the probe by contaminant iron from the medium. After washing with DMEM-HEPES medium and permeabilization buffer (PB- 120 mM KCl, 5 mM phosphate buffer, Eagle's MEM-amino acids mix diluted 1∶500, 10 mM HEPES, 1 μM CaCl2, 1 mM MgSO4, pH 7.2), the cells were permeabilized in PB with 25 μM digitonin for 30 s. The permeabilized cells were washed with PB and taken, in PB containing 2 mM succinate and 1 μM DFO to fluorescence microscopy measurements using a Zeiss Axiovert 35 microscope (Carl Zeiss Inc, North America) attached to a Polychrome V image system (Till Photonics, Munich, Germany). RPA (560 nm excitation-610 nm emission) [20], as described elsewhere [21]. DFO (1 μM) was present in all solutions during permeabilization and fluorescence measurements, to prevent RPA quenching by contaminant iron. After a 4-min baseline was recorded, 0, 5, 10 or 20 μM NAF-1 or 20 μM H114C mutant was added and changes in fluorescence were recorded for 18 min at 37°C followed by 5 μM addition of FHQ (Sigma-Aldrich), so as to attain maximal quenching. Four independent sets of data were obtained and the average from these four sets is shown in the plot of fluorescence versus time. The sequence of fluorescence images acquired by microscopy was analyzed using the Image J program (National Institutes of Health).

## Results

### Cluster transfer from NAF-1 to an acceptor protein

**Figure 2:**
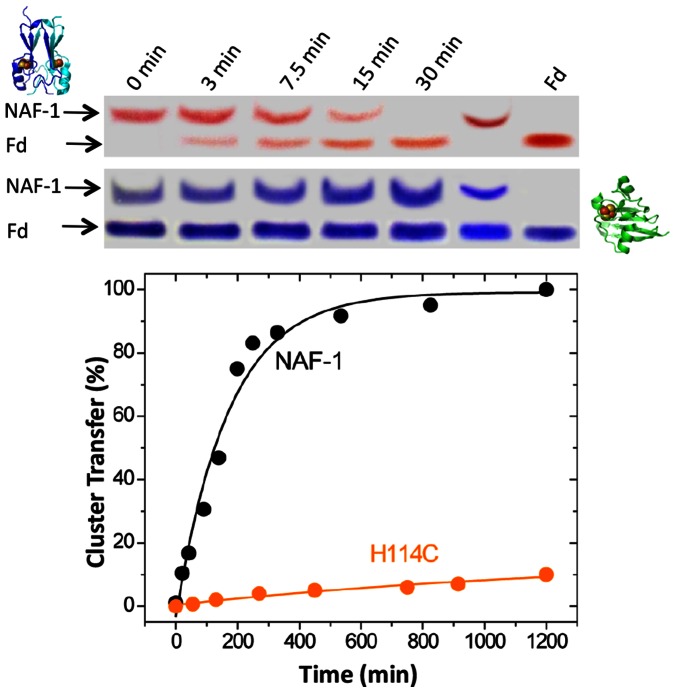
Transfer of NAF-1's [2Fe-2S] clusters to the apo-acceptor protein ferredoxin (apo-Fd). *Upper panel*. NAF-1 was incubated at 37°C with a DTT reduced apo-Fd for increasing times (0, 3, 7.5, 15, and 30 minutes) and the products were run on a native gel. The red colored bands are indicative of the [2Fe-2S] cluster in the two proteins. The diagrams to the left show the structure of the [2Fe-2S] protein NAF-1 (upper left) and the structure of the [2Fe-2S] protein Ferredoxin (lower right) (indicated as holo-Fd with the [2Fe-2S] cluster bound). Replacement of single-coordinating His114 in NAF-1 with Cys (H114C) shows no transfer to apo-Fd after 60 min (labeled as H114C 60 min). In addition, holo-Fd was run as a reference (lane on the far right). A Coomassie stain of the native gel directly below shows similar protein levels in the period of experimentation. *Lower panel*. The percent cluster transfer (CT%) from either wild type NAF-1 or H114C mutated NAF-1 to apo-Fd was determined from UV-Vis absorption spectroscopy data obtained by on-line follow up of the transfer reaction at 458 nm (holo NAF-1 absorbance peak) and at 423 nm (holo-Fd absorbance peak) and calculations as described in experimental procedures. The WT data was fit to an exponential rise as the kinetics display catalytic behavior. The H114C data was fit to a line as the reaction was very slow compared to the WT.

In order to determine if NAF-1 is endowed with cluster transfer ability as is mitoNEET [17] we used pre-reduced apo-Fd as a classical acceptor protein and NAF-1 aa. 57–135 water-soluble domain as cluster donor. The protein profiles obtained by native PAGE (Fig. 2, *Upper panel*) showed a gradual fading of the red NAF-1 and concomitant appearance of red holo-Fd over time, indicating [2Fe-2S] cluster transfer from NAF-1 to apo-Fd with no changes in the respective protein levels, as shown by Coomassie Blue staining. The progress of the transfer to apo-Fd was assessed spectrophotometrically and is depicted in Fig. 2 *Lower panel* as % of cluster transfer CT (%) with time of incubation (illustrative absorption spectra of apo-Fd, NAF-1 and their interaction are given in Fig. S2). The contribution of the *his114* to cluster lability was assessed by replacing *his* to *cys* in NAF-1 at position 114 (H114C mutant in Fig. 2). This change in the cluster binding domain essentially stabilized the [2Fe-2S] cluster and markedly reduced its ability to transfer the 2Fe-2S cluster.

### Cluster transfer dependence on the redox state of NAF-1 and of the acceptor

**Figure 3:**
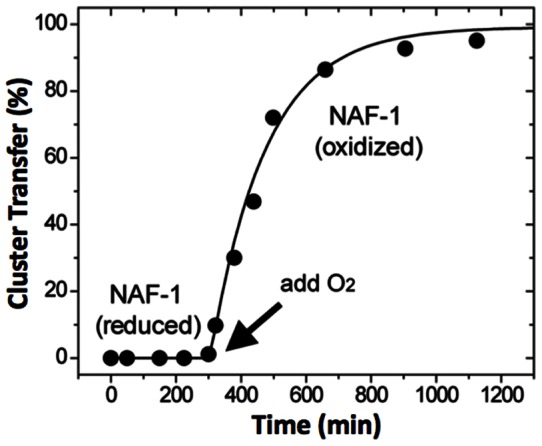
Transfer from NAF-1 only occurs for the oxidized state of the cluster to the reduced state of apo-Fd. *Upper panel*. NAF-1 pre-reduced with dithionite is incapable of transferring its cluster to apo-Fd as analyzed by UV-Vis absorption spectroscopy. Oxidation of the cluster upon addition of oxygen promoted cluster transfer.

For assessing NAF-1chemical requirements for cluster donation/transfer, we first determined spectrophotometrically that transfer proceeds only when NAF-1 was pre-exposed to oxidizing conditions (Fig. 3) and apo-Fd to reducing conditions. No transfer was observed when NAF-1 clusters were reduced by dithionite pretreatment, while exposure to ambient oxygen led to oxidation of the cluster and concomitant transfer of the 2Fe-2S cluster (Fig. S2). The transfer kinetics (Fig. 3) provided an estimated initial rate of 136±20 M^−1^ min^−1^, similar to that found with mitoNEET [17] or for other ISC proteins [22].

**Figure 4:**
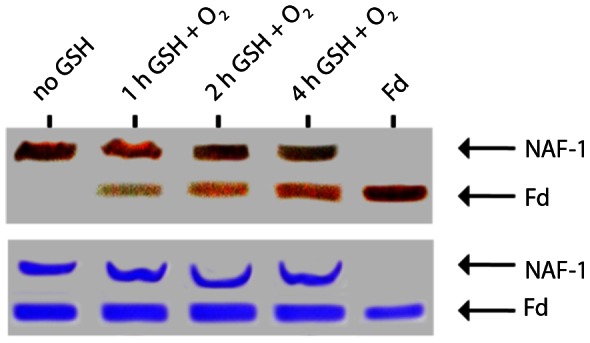
Cluster transfer can occur in the presence of the biological reducing agent glutathione. It is essential to know whether cluster transfer can occur in the presence of the biological reducing agent glutathione. Therefore cluster transfer assays were performed in vitro at 37°C in the presence of 5 mM glutathione (GSH) for the specified length of time and then analyzed by Native PAGE. Fd with its [2Fe-2S] cluster (holo-Fd) was run as a reference. The upper gel is not stained and shows the transfer of the [2Fe-2S] cluster by the visible red bands on the gel. The lower panel is the same gel after staining with Coomassie.

In order to better mimic cellular conditions, we subjected the proteins to the more physiological reducing agent glutathione (GSH) [23], which is found in cells at ∼5 mM and together with NADPH contributes to cell redox capacity [24]. We found that GSH (but not GSSG) at physiological concentrations can activate the apo-acceptor and significantly enhance transfer over time (Fig. 4). Hence cluster transfer is indeed viable under cellular conditions.

### Iron transfer from NAF-1 to intact mitochondria

**Figure 5:**
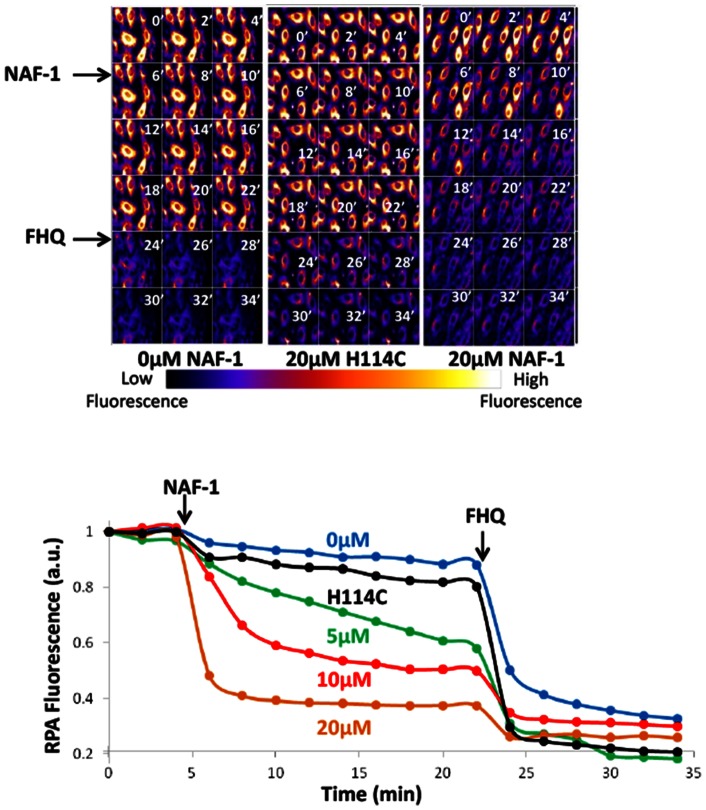
Transfer of labile iron from NAF-1 to mitochondria. *Upper panel*. Pseudo-colored images of permeabilized h9c2 cells labeled with red rhodamine B-[(1,10-phenanthrolin-5-yl)] benzyl ester (RPA) to trace iron in the mitochondrial matrix and then measured for fluorescence every two minutes. NAF-1 (WT or H114C mutated) was added to either 0, 5, 10, or 20 μM concentrations after 4 minutes (only 20 μM profiles are shown). The pseudocolor of the cells indicates the relative levels of mitochondrial RPA fluorescence (orange: high; blue: low). The liposoluble permeant FHQ, was added to 5 μM after 22 min in order to attain maximum quenching. *Lower*. Plot of RPA fluorescence is given in terms of arbitrary units (a.u.) obtained by analyzing individual cell fluorescence with Image J as described in Experimental Procedures. The RPA fluorescence is the average of four independent runs.

The cluster transfer ability of NAF-1 was also assessed in mammalian cells using mitochondria as a potential organellar acceptor of labile iron. We used for that purpose cells double fluorescently labeled with iron sensors [25]: red rhodamine B-[(1,10-phenanthrolin-5-yl)] benzyl ester (RPA) for tracing labile iron in the mitochondrial matrix [20], and calcein-green (CALG) for tracing labile iron in the cytoplasm [20]. To render mitochondria accessible to NAF-1 we first permeabilized the cells by a protocol that preserved the mitochondria intact and functional, as they retained the potentiometric RPA iron probe while losing most of the cytosolic calcein [21]. Fig. 5 (*Upper panel*) depicts fluorescence images taken over time following addition of either NAF-1 (only 0 µM and 20 µM profiles are presented) or H114C mutated NAF-1 (20 µM). Fig. 5 (*Lower panel*) depicts the time dependent changes in RPA fluorescence intensity per cell analyzed from fluorescence images as those depicted in Fig. 5 (Upper panel). Both Fig. 5 *upper & lower panels* show that addition of NAF-1 to RPA labeled cells evoked a time dependent quenching of mitochondrial RPA fluorescence, indicating labile iron transfer from NAF-1 to the mitochondrial matrix, where RPA is highly localized [18],[25]. The transfer of labile iron was concentration dependent in the 0–20 µM range of wt NAF-1, whereas application of the H114C-mutated NAF-1 failed to evoke a detectable cluster transfer to mitochondria even at the highest concentrations used. In the present series of studies (Fig. 5 *upper & Lower panel*) we have used a membrane-permeant complex of labile iron, FeCl_3_-8-hydroxyquinoline (FHQ), as an experimental tool to estimate the maximum attainable quenching of mitochondria RPA fluorescence [18].

### NAF-1 cluster and iron transfer abilities' susceptibility to antidiabetic drugs

**Figure 6:**
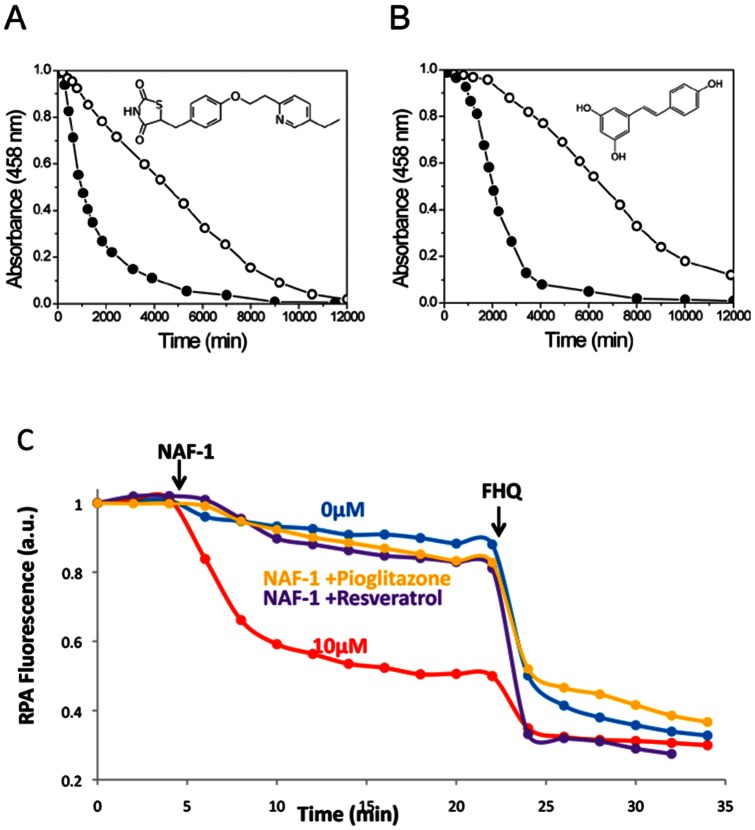
Effect of pioglitazone and resveratrol on NAF-1: cluster stabilization and abrogation of iron transfer to mitochondria. *Upper panel.* Kinetics of NAF-1 stability in the absence and presence of pioglitazone monitored spectrophotometrically (458 nm) at 37°C at pH 7.0 (NAF-1 and pioglitazone 20 µM each). The half-decay time of the absorbance corresponding to NAF-1 (2Fe-2S) cluster was raised by pioglitazone from t_1/2_  = 1000±160 min (filled circles) to t_1/2_  = 4700±350 min (open circles). Likewise, 20 µM resveratrol delayed NAF-1 cluster decomposition with a t_1/2_  = 6800±500 min (open circles). *Lower*. Effect of drugs on NAF-1 ability to transfer labile iron to RPA labeled mitochondria using permeabilized h9c2 cells. Data acquisition, analysis from fluorescence images and plotting were done as described in the legend to Fig. 5. Addition of NAF-1 (10 μM) to RPA-labeled permeabilized cells at 4 min generated a fast quenching of RPA (red) whereas addition of none (blue) was steady until supplemented with the permeant FHQ (5 μM) at 22 min, which led to maximal attainable quenching. The addition of NAF-1 preincubated with resveratrol (20 μM) (purple) or pioglitazone (yellow) abrogated cluster transfer as evidenced by the absence of RPA fluorescent quenching, again, until the system was challenged with the permeant FHQ.

The fact that NAF-1 shares similar structural motifs and biophysical properties with mitoNEET which in turn shows susceptibility to the antidiabetic pioglitazone [2],[13], led us to consider NAF-1 as a potential target of TZD drugs. Moreover, computational docking simulations [26],[27] of small molecules to CISD family members yielded the TZD drugs pioglitazone and rosiglitazone and the longevity-promoting natural product resveratrol as potentially high-affinity binders partners [26]. These properties led us to comparatively assess pioglitazone effects on NAF-1 vs. mitoNEET using the above-mentioned assays. The data shown in Fig. 6 essentially recapitulated key findings obtained earlier with mitoNEET [17] in terms of blockage of both spontaneous cluster decomposition (Fig. 6A&B) and cluster transfer to RPA-labeled mitochondria (Fig. 6C). The addition of pioglitazone to NAF-1 at pH 7.0, where the [2Fe-2S] clusters are pH-labile, led to almost 5- fold cluster stabilization, as it raised the t_1_/_2_ of cluster loss from 1000±160 min to 4700±350 min (Fig. 6A). Significantly, incubation of NAF-1 with the anti-diabetes drug pioglitazone prior to addition to RPA labeled cells (as seen in Fig. 5) abrogated the change in RPA fluorescence evoked by NAF-1 (Fig. 6C). This novel finding identifies NAF-1, the human paralog of mitoNEET [17], as the possible second mitochondrial target of TZD drugs, clarifying the puzzle proposed recently [13]. We also tested the ability of resveratrol to bind the protein NAF-1 and found it raised the t_1_/_2_ of spontaneous decomposition from 1000±160 min. to 6800±500 min. The attainment of a 7 fold cluster stabilization is akin to that observed above as well as with the TZD drug pioglitazone on mitoNEET [2]. Moreover, the natural product resveratrol also abrogated the cluster transfer from NAF-1 to mitochondria (Fig. 6C), indicating that also in cells it acted on NAF-1 similarly to the TZD pioglitazone. Interestingly, resveratrol evoked no detectable stabilization of the mitoNEET [2Fe-2S] cluster over the pH range of 6.0–7.5 (data not shown).

## Discussion

We have identified NAF-1, which is known to play a role in inhibiting autophagy [10] and promoting longevity [7],[8] and whose cellular absence leads to numerous pathologies [9] as an [2Fe-2S] cluster transfer protein (Fig. 2). It's cluster transfer capabilities occur only when the cluster is oxidized [2Fe-2S]^2+^ and the apo-acceptor is reduced by chemical reducing agents or more importantly by physiological agents such as glutathione (Fig. 3 and 4). While its backbone structure is similar to mitoNEET, and it shares some functional and pharmacological properties [1],[2],[17], NAF-1 has a distinct protein surface (Fig. S1) and is distinct in its involvement in health and disease. Nonetheless, it shares the binding of the anti-diabetes type II drug, pioglitazone, originally used to identify mitoNEET as a major cellular target [11]. The TZDs were recently suggested to act also on additional mitochondrial component, as binding still occurred in mitoNEET animal knockout [13]. In the present work we provide a positive identification of NAF-1 as a target of pioglitazone, and an important potential physiological target of TZDs in mitochondria. Moreover we show that TZD drug binding abrogates cluster transfer capacity from NAF-1 to acceptor proteins and to mitochondria (Fig. 6). This finding might have physiological and pharmacological implications for various disorders in which NAF-1 has been implicated [9]. Following that lead we experimentally verified that resveratrol can indeed stabilize NAF-1 (but not mitoNEET) [2Fe-2S] cluster and thereby also interfere with its transfer to mitochondria (Fig. 6). This finding underscores the importance that small molecules that differentially bind to mitoNEET or NAF-1 will help elucidate the likely multifunctional role of these proteins in health and diseases, a task we are actively pursuing.

## Supporting Information

Figure S1 **Structural comparison of NAF-1 and mitoNEET.** (*A*) Ribbon diagrams of the soluble parts of NAF-1 and mitoNEET (amino acids 57–135 and 33–108 respectively) derived from published X-ray analyses (1,2,1) highlighting the aromatic surface residues. The differences in the side chain composition lead to differences in the surface of the two proteins, a feature that results in selective binding of resveratrol and other potential small therapeutic molecules. (B) NAF-1 and mitoNEET amino acid sequences: letters in green denote the water soluble C-terminus, those in black denote the trans-membrane segment, and the soluble part facing the cytoplasm is shown in pink. The symbols denote the level of similarity between the amino acids: asterisk, identity; colon, high similarity; period, poor similarity; no sign, no similarity. The blue line above the sequence corresponding to the soluble portion of the sequence indicates secondary structure elements: arrow for beta sheet and a thick line for alpha helix. The thin lines indicate no secondary structure. (C) Aromatic side chains superimposed on the backbone structures. Note that the side chains, reflective of the protein surface, are significantly different.
(TIF)

Figure S2 **NAF-1 transfers its cluster only when its [2Fe-2S] clusters are in the oxidized state.** (A) Apo-Fd is preincubated with DTT to ensure the availability of the cysteine side-chains of the acceptor protein for cluster coordination. Under these conditions NAF-1 is reduced and cluster transfer is inhibited as evidenced by the absence of any changes in the NAF-1 Visible spectra over time. The spectrum of apo-Fd shows complete loss of cluster (aqua, <0.05 absorbance units). (B) After 300 min, oxygen is added to the solution and cluster transfer proceeds readily. The visible spectra show that NAF-1 is quickly oxidized upon addition of oxygen (black) and the cluster is transferred to apo-Fd over time (blue, red). The visible trace of apo-NAF-1 shows complete loss of cluster (grey, <0.05 absorbance units).
(TIF)
