# Supplementary figures and images for: Nutrient-Deprivation Autophagy Factor-1 (NAF-1): Biochemical Properties of a Novel Cellular Target for Anti-Diabetic Drugs
Source: PLoS One. 2013 May 22;8(5):e61202. doi: 10.1371/journal.pone.0061202 (PMC3661554; doi:10.1371/journal.pone.0061202)

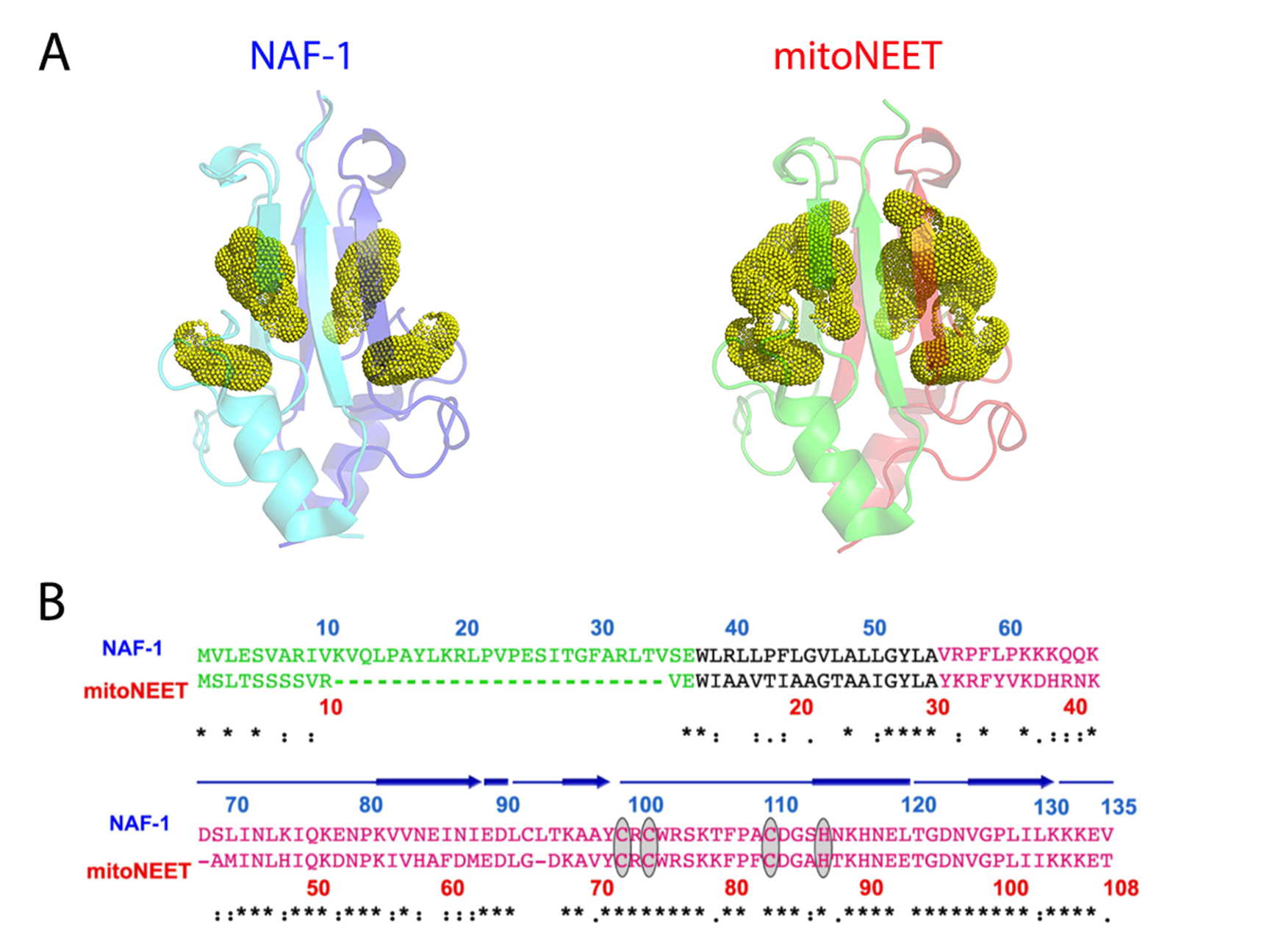

Supplement: Figure S1 — Structural comparison of NAF-1 and mitoNEET. (A) Ribbon diagrams of the soluble parts of NAF-1 and mitoNEET (amino acids 57–135 and 33–108 respectively) derived from published X-ray analyses (1,2,1) highlighting the aromatic surface residues. The differences in the side chain composition lead to differences in the surface of the two proteins, a feature that results in selective binding of resveratrol and other potential small therapeutic molecules. (B) NAF-1 and mitoNEET amino acid sequences: letters in green denote the water soluble C-terminus, those in black denote the trans-membrane segment, and the soluble part facing the cytoplasm is shown in pink. The symbols denote the level of similarity between the amino acids: asterisk, identity; colon, high similarity; period, poor similarity; no sign, no similarity. The blue line above the sequence corresponding to the soluble portion of the sequence indicates secondary structure elements: arrow for beta sheet and a thick line for alpha helix. The thin lines indicate no secondary structure. (C) Aromatic side chains superimposed on the backbone structures. Note that the side chains, reflective of the protein surface, are significantly different. (TIF) [file pone.0061202.s001.tif]

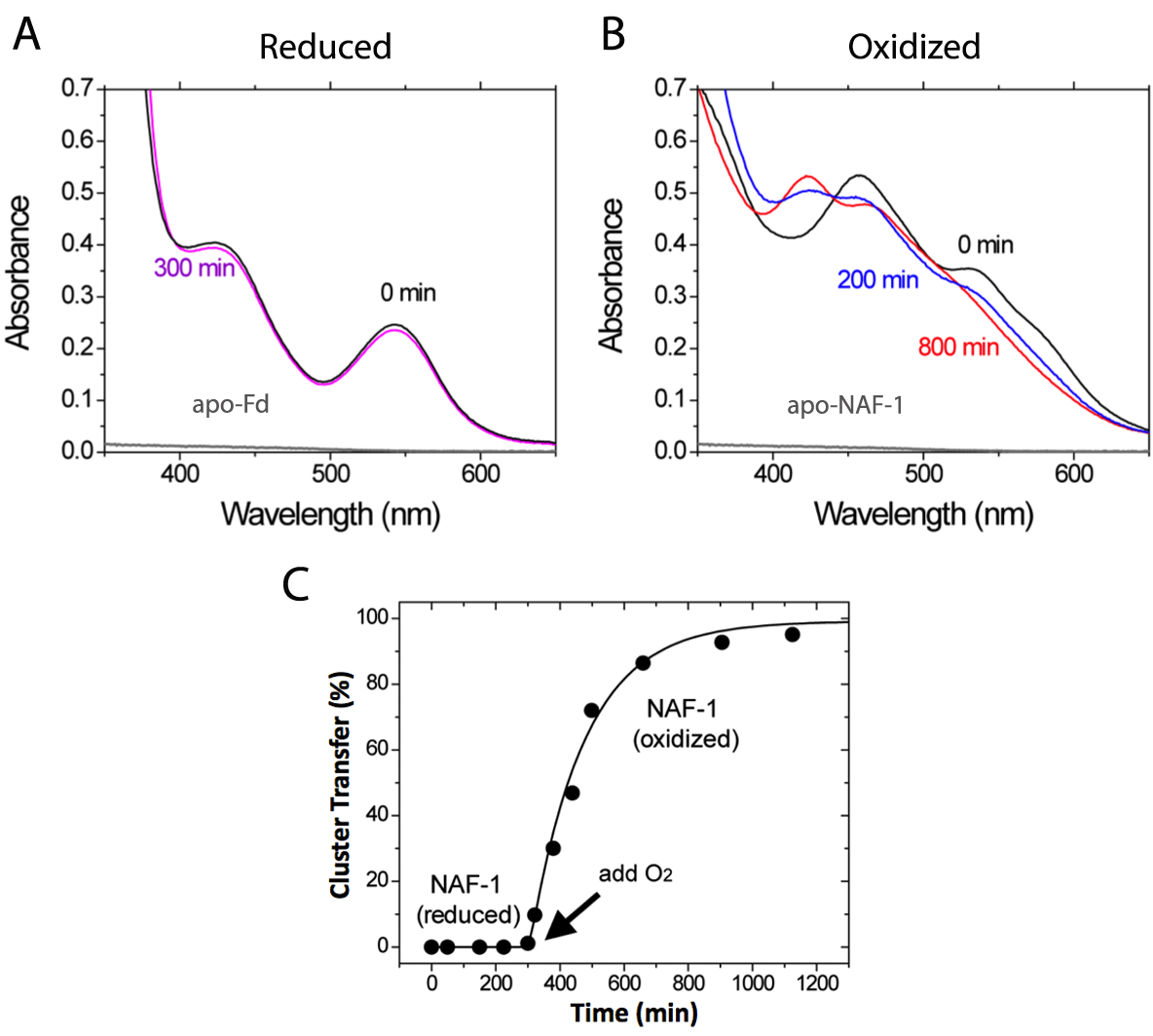

Supplement: Figure S2 — NAF-1 transfers its cluster only when its [2Fe-2S] clusters are in the oxidized state. (A) Apo-Fd is preincubated with DTT to ensure the availability of the cysteine side-chains of the acceptor protein for cluster coordination. Under these conditions NAF-1 is reduced and cluster transfer is inhibited as evidenced by the absence of any changes in the NAF-1 Visible spectra over time. The spectrum of apo-Fd shows complete loss of cluster (aqua, <0.05 absorbance units). (B) After 300 min, oxygen is added to the solution and cluster transfer proceeds readily. The visible spectra show that NAF-1 is quickly oxidized upon addition of oxygen (black) and the cluster is transferred to apo-Fd over time (blue, red). The visible trace of apo-NAF-1 shows complete loss of cluster (grey, <0.05 absorbance units). (TIF) [file pone.0061202.s002.tif]
